# Practical Sanitation

**Published:** 1885-08

**Authors:** 


PRACTICAL SANITATION.

The following extract is from instructions recently issued by the Health Commissioner of New York city, as published in *The Sanitarian:*

DIPHTHERIA, scarlet fever, measles, and small-pox are highly contagious diseases, attacking persons of all ages, and may be contracted from those who are already affected, from the clothes that they have worn, and from everything which has been in the room with them. Even the walls of the room may be a source of infection to persons coming into it after the patient has left it, unless the infectious material is destroyed. In order to prevent the spread of these diseases in a family or house where they exist, and to promote the recovery of the sick, the following simple measures should be conscientiously and rigidly carried out, thereby preventing much suffering and saving human life.

An upper, sunny room, provided if possible with an open fireplace, and with no children on the same floor, should be arranged for the patient by removing everything from it which can possibly be spared, such as books, clothing, carpets, upholstered furniture, and window-curtains; also plants, birds, and other pets, remembering that when once the patient has entered the room nothing can with safety be removed until disinfected. By thus stripping the room of all articles except those absolutely necessary, the subsequent disinfection is much more easily performed. If it is deemed necessary, a few small rugs will take the place of the carpet.

The fireplace serves a double purpose: first, as a means of ventilation; and second, by keeping a small fire burning therein, when the weather will permit, the pieces of soft muslin or other material, which should always be used instead of towels or handkerchiefs in wiping the secretions from the mouth or nose, especially in diphtheria, can readily be destroyed by fire, and thus contagion through them prevented.

One or two adults should take the entire charge of the patient, under no circumstances coming in contact with other persons, especially children. Kissing and "taking the breath" of persons having contagious disease are especially dangerous, and should always be avoided. Open windows and open fireplaces, with fire in them day and night, avoiding draughts and chilly air, protect the sick and those who nurse them.

Nothing should be removed from the room when the patient has once entered it until it has been thoroughly disinfected.

Books, scrap-books, toys, or other playthings should always be destroyed at the termination of the sickness, as being undoubted carriers of contagion. Locks of hair and other keepsakes have also been known to spread contagious disease.

Nurses should keep themselves and their patients as clean as possible, remembering that the more the infection accumulates the more dangerous does it become. Special care should be taken in changing sheets and the clothing not to shake them or disturb them more than is absolutely necessary to remove them. As these acts disseminate the particles of skin which are removed with them, and which convey the germs of the disease, they should be removed carefully and folded together, and immediately disinfected.

DISINFECTION.

It is a popular idea that anything which destroys an offensive odor is a disinfectant. This is not only erroneous, but harmful, as reliance is thus placed on substances that in nowise act as destroyers of infectious material, which latter substances are the only true disinfectants. The methods recommended in this circular are, to a considerable extent, based upon the results of the work of the Committee on Disinfectants of the American Public Health Association.

DISINFECTANTS.

The agents recommended herein for disinfection are:

1. Fire.

2. Boiling water.

3. Chloride of lime or chlorinated lime, either dry or in solution, as Standard Solution No. 1.

4. Solution of chlorinated soda, diluted as Standard Solution No. 3.

5. Sulphur.

6. Bichloride of Mercury.

Bichloride of mercury, or corrosive sublimate, a powerful disinfectant, is included in the above list for one purpose only; that is, for the disinfection of privy-vaults which contain a large amount of material believed to be infected. As this circular is intended for general distribution, the writer hesitates to recommend for general use an agent which may, through improper use, endanger life.

*Fire.*—As already directed, the materials used in wiping away the discharges of the sick may be burned in the open fireplace, if such there be. In general, this method of disposal is to be recommended for all substances which have been exposed to infection, which cannot be treated with boiling water, and could it be carried out in all cases, would make disinfection a very simple matter. If it is desired to burn substances suspected of being infected, and there is no fire in the room, such substances may be wrapped in a sheet soaked with Standard Solution No. 3, hereafter referred to, and in this condition conveyed to the fire in the furnace or elsewhere.

*Boiling Water.*—Experiment has demonstrated that boiling in water for half an hour will destroy the vitality of all known disease-germs. This is therefore recommended as the best means to be employed in the disinfection of all articles which can be thus treated, such as the body-clothing of the patient, the bedclothes, towels, etc. All utensils which are used in the room in the feeding of the patient, such as plates, tumblers, spoons, knives, forks, etc., should likewise be treated with boiling water before being removed from the room. Food itself not consumed by the patient should not be used by others, as it is liable to become infected in the sick-room.

If, as will often be the case, there are no facilities for treating articles with boiling water in the sick-room, they may with safety be removed to another part of the house for this treatment if they are carefully enveloped in a towel or sheet, as the case may require, which has been thoroughly soaked with either Standard Solution No. 1 or Standard Solution No. 3. Thus enveloped, they should be put in the water, and boiled for the required time.

*Chloride of Lime.*—This substance, also called chlorinated lime, to be effective as a disinfectant, must be of the best quality, and in purchasing it only that should be accepted which is enclosed in glass bottles, as when packed in paper or wooden boxes it is liable to have so deteriorated as to be worthless for disinfecting purposes. When dissolved in water, in the proportion of four ounces to the gallon, it forms the Standard Solution No. 1, recommended by the Committee on Disinfectants. The solution thus prepared is to be used in the disinfection of discharges in contagious diseases, especially in typhoid fever and cholera. One pint should be well mixed with each discharge; after ten minutes disinfection is completed, and the contents of the vessel may be then safely thrown in the privy vault or water-closet. The expectorated matter of those sick with consumption should be discharged into a cup half filled with this solution or with Standard Solution No. 3.

To thoroughly disinfect a privy vault, Standard Solution No. 1 should be used in large quantities—one gallon for each gallon of material in the vault; the surface of the contents should be subsequently daily covered with the dry chloride of lime. The cost of the Solution No. 1 is about three cents a gallon.

*Solution of Chlorinated Soda.*—To be effective, this solution must contain at least three per cent. of available chlorine, and in purchasing it care should be exercised to obtain such a quality. This is sometimes spoken of as Labarraque's Solution; but as this latter substance is too weak to act as a disinfectant, the name is liable to mislead, and is therefore here not used. The Standard Solution No. 3 of the committee is made by adding five parts of water to one part of the solution of chlorinated soda. The cost of this solution is about ten cents a gallon. When thus diluted, it may be used for all the purposes for which Standard Solution No. 1 was recommended, and is of a somewhat more agreeable odor, though more expensive.

This solution should be used to cleanse portions of the body soiled with discharges of those sick with infectious diseases, or the hands of attendants similarly soiled

*Bichloride of Mercury* (Corrosive Sublimate), is recommended in this circular to be used only in the disinfection ot privy-vaults which contain so much material, believed to be infected with the germs of typhoid fever or cholera, that the disinfection by Chloride of Lime would be impracticable. In using this it should be dissolved in the proportion of one ounce of Bichloride of Mercury to one gallon of water; this quantity will disinfect four gallons of infected excremental matter.

TREATMENT OF THE BODY OF THE PATIENT AFTER RECOVERY OR DEATH.

When the patient has recovered, he should be first sponged over with the solution of chlorinated soda, diluted in the proportion of one part to twenty parts of water; and, indeed, during the course of the illness, occasional sponging of the body with this very diluted solution, under the direction of the attending physician, will be of value in preventing the escape from the surface of the body of infectious material. When, after recovery, the body has been thus sponged, not omitting the head and hair, a thorough washing of the body with soap and warm water should follow, and the patient dressed in clothes which have not been exposed to infection. This should take place in another room than the one occupied during the illness.

Should the case result fatally, the body should be thoroughly sponged with either Standard Solution No. 1 or No. 3, and then wrapped completely in a sheet saturated with one of these solutions, and enclosed in a coffin, which is to be closed, and the interment must take place within twenty-four hours, and be strictly private. If the interment is to take place at a distance, requiring transportation by any other means than a hearse, the coffin must be of metal, or metal-lined, and hermetically sealed.

